# Lectures on the Diagnosis and Treatment of Diseases of the Lungs

**Published:** 1841-01-09

**Authors:** W. W. Gerhard


### MEDICAL EXAMINER.

DEVOTED TO MEDICINE, SURGERY, AND THE COLLATERAL SCIENCES.

No. 2.] PHILADELPHIA, SATURDAY, JANUARY 9, 1841. [Vol. IV.

### LECTURES ON THE DIAGNOSIS AND TREATMENT OF DISEASES OF THE LUNGS.

BY W. W. GERHARD, M. D.

### LECTURE XII.

GANGRENE.

I have now gone through pneumonia in all its forms, and shall proceed to the consideration of an affection, which, though not very frequently met with, still requires a notice in this place: I refer to gangrene of the lungs. This, like gangrene in other parts of the body, may occur either as a primary or secondary affection. When primary, it is probably owing to an alteration in the condition of the blood, which, being rendered unfit for nutrition, can no longer support the vitality of the parts. It occurs as a secondary affection in cases of asthenic pneumonia. The anatomical characters of the gangrene are nearly the same in both forms, although, when it is in its secondary form, the tissue is at first hard and congested, and is seated in the midst of an inflamed parenchyma, while, in the primary form, it is merely infiltrated with a thin, serous liquid, which is evidently in a state of incipient gangrene, and gives rise to the fœtor of the breath met with even in the first stage of the affection.

In the second stage, the tissue begins to break down, and gangrenous matter is expectorated; next, the bronchial tubes slough off, and nothing is left in a sound state but the vessels: these resist the destructive process for a long time; and on examination after death they are usually seen traversing the cavity; however, after a while, they, too, are destroyed, and their destruction sometimes gives rise to a hæmorrhage which destroys the patient, although generally the blood has ceased to circulate through them before they slough, and little or no hæmorrhage ensues. The sputa and breath in this stage of the disease are pathognomonic; they are both exceedingly fœtid, and the disease can, on this account, be easily distinguished from any other. There are two varieties of the gangrenous sputa: one consists of a dark thin liquid, which somewhat resembles tobacco juice, or liquorice, occasionally containing small pieces of black, gangrenous lung; the other consists of a grayish-yellow pasty fluid, which is probably a mixture of pus and gangrenous liquid; the latter occurs most frequently in cases following pneumonia; both, however, are extremely fœtid, though the odour differs slightly. In some cases of phthisis the sputa resemble the second variety, and it is probable that in these cases the tuberculous portion of the lung becomes gangrenous.

The third stage begins with the formation of a cavity, which continues to increase for some time, and may go so far as to involve a lobe, or even nearly the whole of one lung. After the formation of the cavity, the sputa are nearly the same, consisting of a thin, fœtid liquid, frequently stained with blood, which flows from the sphacelated vessels. When the case terminates fatally, the sputa increase in quantity, and the patient gradually sinks until he is completely exhausted, and death ensues. But when the disease terminates favourably, the following changes take place: the gangrenous portion of the lung is first circumscribed by a membrane which separates it from the surrounding healthy tissue. As the gangrenous portion sloughs away, this membrane is left as a lining to the cavity, and secretes pus; therefore, we find the latter fluid at first mixed with the gangrenous sputa, and supplanting it entirely when the whole of the diseased portion has been removed. As the inflammation subsides, the membrane assumes the character of a mucous membrane, and at last becomes similar to that lining smaller tubes and air-vesicles, which resembles very closely the serous membranes in the delicacy of its texture. If the cavity is excluded, the lining membrane being no longer exposed to the stimulus of the air, loses its mucous character entirely, and we find a cyst lined with a membrane, which is almost serous, and nearly similar to that found in the brain and elsewhere after cicatrization; this may continue during the remainder of the existence of the individual, or be gradually obliterated by the formation of cellular tissue.

After the entire cure of the gangrene, the tissue involved is more or less dense, and contains less than the natural proportion of air.

The local signs of this disease are the cough, expectoration, and fœtor of breath. The cough at first resembles that of ordinary catarrh, but as the disease advances, it becomes looser and paroxysmal in its character, which is produced by the accumulation of fluid in the bronchial tubes requiring a violent effort to throw it off, which ceases as soon as this is accomplished, and the paroxysm does not recur until the accumulation of fluid again renders this effort necessary. These fits of coughing are often extremely distressing to the patient.

The physical signs are, in the first stage, feeble respiration and a moist rhonchus, generally either the mucous or sub-crepitant; the percussion is either natural or a little dull. They are not, therefore, characteristic.

As the disease advances, we find the usual signs of a cavity, viz.: cavernous respiration, a loose gurgling and cavernous resonance of the voice, or pectoriloquy; the last, however, is not so clear as in phthisis, unless the cavity should be large, and near the surface of the lung, for the quantity of liquid in the cavity, and the softness of its parietes, deaden the resonance. When cicatrization takes place, we find merely feebleness of respiration, which gradually diminishes, but does not entirely disappear. If the liquid is discharged from the cavity in its early stages, the cavernous respiration and resonance of the voice are rendered much clearer.

The general signs are the following: there is usually considerable fever during the progress of the disease, with a small, frequent, irritable pulse; sometimes the pulse is exceedingly feeble. The fever is only important as it is connected with the prognosis, which is very unfavourable when the fever is high, and the gangrene is progressing; but if the disease do not advance, the fever is unimportant. There is an almost complete loss of appetite, produced by the nauseating character of the gangrenous liquid which is swallowed by the patient, who often has diarrhœa from the same cause. The skin is pale, and usually lead-coloured in the advanced stage, which is observed in almost all cases of gangrene, whatever part of the body may be affected. Very often there is extreme dyspnœa.

*Prognosis.*—As an ordinary result, about one-half of those attacked will die. In hospitals the mortality is rather greater, amounting to three-fifths, while in private practice it is probably about two-fifths.

*Diagnosis.*—The only pathognomic characters of gangrene, are the fœtid breath, and expectoration of the patient. When these occur as an acute disorder, or supervene suddenly upon a chronic one, they are quite characteristic of the disease. If they occur slowly, and continue for a long period, they depend upon a vitiated secretion of the bronchial membrane, caused by chronic bronchitis; but this either never occurs in acute inflammations of the lungs, or is so rare as not to be taken into the account. Numerous as are the cases of gangrene which I have met with in hospital practice, I do not recollect a case in which the fœtid sputa arose from simple acute bronchitis. The other signs of the disease are common to it and some other affections of the lungs; but the rapidity of the softening, and the formation of a large cavity in a short period, occur so seldom except from gangrene, that these signs are very good indications of the disease.

*Causes.*—About these it will be proper to say a word or two before going farther. The proximate, and at times mainly predisposing cause of this affection, is an altered condition of the blood, which becomes thin, and probably is vitiated in some unknown manner, which frequently coincides with a local inflammation. The ultimate causes are intemperance, indulgence in food of an unnutritious nature. An attack of some acute disease, most frequently pneumonia, is the immediate exciting cause in rather more than half the cases: in others, the disease is general, and arises from the fluids alone. In both cases gangrene of the lungs at times follows that of other parts of the body.

*Treatment.*—This is not in most cases antiphlogistic, but supporting in its character, tonics and stimulants being required. When you detect the occurrence of gangrene, you must use all the means that you possess to support the strength of the patient, who is in a short time very much prostrated; for this purpose you must administer stimulants and tonics, with the free use of porter, wine, and nutritious food. This is the best and almost only mode of treatment. There is a remedy, however, which I have used in addition, and, I think, with some benefit, viz. chlorine; I give from ten to twenty drops of the solution of the chloride of soda every three or four hours; if, however, there is disposition to diarrhœa in the patient, he will bear very little of it. In addition to the internal use of chlorine, I place near the patient's bed, vessels containing chloride of lime, which adds much to the comfort of the patient and his attendants. Opium is necessary in some cases of gangrene of the lungs, to check the violent paroxysms which return so frequently as to fatigue the patient extremely; but it should be given sparingly, for it has the disadvantage of checking the secretions of the lungs; hence, it should be administered in the smallest possible quantity, and even then may be combined with senega and ipecacuanha, unless the nausea should be excessive.

The indications for the treatment of gangrene are, therefore, extremely simple; a generous, supporting diet and treatment, with blisters, and, in a few cases, cupping to the chest, to check the intercurrent and accompanying inflammation, constitutes our main reliance, but the chances of success are greatly increased by the accessary remedies, some of which I have mentioned. The absolute antiphlogistic treatment is decidedly bad, and of the remedies which are classed under this head, none is more positively mischievous than mercury and its various preparations.

